# Regional cutaneous and muscle sensitivity does not mediate changes in active mouth opening in temporomandibular disorders: a cross-sectional study

**DOI:** 10.22514/jofph.2025.051

**Published:** 2025-09-12

**Authors:** Jorge Ballesteros-Frutos, Rubén Fernández-Matías, Inmaculada Torres-Tejada, Daniel Pecos-Martín

**Affiliations:** ^1^Physiotherapy and Pain Group, University of Alcalá, 28805 Alcalá de Henares, Spain; ^2^Department of Physiotherapy, University of Alcalá, 28805 Alcalá de Henares, Spain; ^3^Department of Physiotherapy, Doctoral School, University of Alcalá, 28805 Alcalá de Henares, Spain; ^4^Department of Physiotherapy, Doctoral School, University of Valencia, 46010 Valencia, Spain; ^5^Physiotherapy Unit, University Hospital of Guadalajara, 19002 Guadalajara, Spain

**Keywords:** Temporomandibular disorder, Physical therapy, Mediation, Causality

## Abstract

**Background**: One of the main goals of treatment in temporomandibular disorder (TMD) patients is to improve mouth opening range of motion. However, it is not clear which factors influence its alteration. The aim of this study was to compare differences in mechanosensitivity, mouth opening and psychosocial factors, between people with and without TMD, to evaluate if changes observed in active mouth opening are mediated by an increase in tissues’ mechanosensitivity, and to evaluate factors related to mandibular-related disability. Subjects with and without TMD were recruited. **Methods**: Cross-sectional study conducted in Spain. The measured variables were the Craniofacial Pain and Disability Inventory (CF-PDI), pain intensity, pressure pain thresholds (PPT) at local points; active and passive mouth opening; the Short-Form 12 questionnaire (SF-12); the State-Trait Anxiety Inventory (STAI); and the Neck Disability Index (NDI). Multivariable regression and mediation models were constructed. **Results**: A total of 179 subjects (85 with TMD) were included. Subjects with TMD had less mouth opening range of motion, and lower PPT. Tissues’ mechanosensitivity did not mediate the reduction in active mouth opening in subjects with TMD (overall indirect effect, 0.98; 95% confidence interval, −0.87 to 3.12). Finally, no variable showed an association with CF-PDI. **Conclusions**: Subjects with TMD seem to have decreased mouth opening, and greater mechanosensitivity of masticatory muscles, when compared with healthy controls. Tissues’ mechanosensitivity does not seem to mediate the reduction of active mouth opening in subjects with TMD, and there seems to be no relationship between PPT measures, mental health outcomes, the NDI and mandibular-related disability.

## 1. Introduction

Temporomandibular disorder (TMD) is a broad term encompassing a group of musculoskeletal and neuromuscular conditions, that can affect the masticatory muscles, the temporomandibular joint and other related structures, either in isolation or in combination [1]. It is characterized by a wide range of symptoms such as clicking or locking of the jaw, mandibular and neck pain, headaches, tinnitus and fatigue [2, 3, 4, 5]. Among these, pain is the most prominent and prevalent symptom, followed by restricted jaw movement [2], both of which significantly reduce perceived quality of life [3].

Temporomandibular disorder is the most common reason for seeking dental care other than dental pain [5], and it ranks as the second most prevalent musculoskeletal disorder [3]. Its prevalence in the general population is estimated to range between 25% and 35%, with a higher incidence reported among women [4]. During the last few years, more attention has been given to the TMD due to studies showing its association with substantial economic costs and physical, social and emotional burden on affected individuals [4, 5].

The etiology of TMD is not well understood. It is generally considered multifactorial, involving a combination of diverse and heterogeneous factors [4, 5], such as tissue mechanosensitivity [3], anxiety [5], stress [5] and depression [4].

TMD has also been related to neck pain [6, 7], due to the convergence of the trigeminal and cervical neurons afferences in the cervical trigeminal complex of the brainstem [3], integrating nociceptive signals from both regions [2, 4, 8]. Although several studies have investigated the relationship between neck disability and jaw dysfunction, many of them lack appropriate control for potential confounding variables [6].

One of the primary goals in the treatment of patients with TMD is to restore the maximum range of active mouth opening [3, 9]. However, the factors contributing to limitations in this movement remain unclear. Structural abnormalities through imaging are not consistently present in all TMD patients, and similar findings may appear in asymptomatic individuals [10, 11].

Therefore, the decrease in mouth opening range cannot be explained entirely by mechanical factors alone.

Previous investigations have shown that individuals with TMD have increased tissue mechanosensitivity, both locally and in remote areas [6, 12]. It has been proposed that heightened mechanosensitivity, especially in muscles and other tissues innervated by the branches of the trigeminal nerve, may contribute to a decrease in active mouth opening [13].

Mechanosensitivity can be defined as the ability of sensory neurons to respond to mechanical stimuli, such as pressure, stretch or vibration. It can be measured in many ways, one of them is pressure pain threshold (PPT), which measure how much pressure is needed in a body so the sensation changes from pressure to pain, with lower values indicating greater mechanosensitivity [14].

Due to the complexity of TMD, its multifactorial nature [4, 5] and the lack of conclusive evidence regarding effective treatments [3], managing these patients remains a clinical challenge. Furthermore, there is limited understanding of the causal pathways underlying the physical impairments and symptoms associated with TMD [13].

Therefore, the aim of this cross-sectional study is threefold: (1) to comprehensively compare mechanosensitivity, active mouth opening, and psychosocial factors between individuals with and without temporomandibular disorders (TMD), in order to enhance our understanding of the multifactorial nature of TMD and the associated physical and psychological impairments; (2) to evaluate whether changes in active mouth opening are mediated by increased tissue mechanosensitivity, thereby shedding light on potential mechanisms involved in the limitation of mandibular function; and (3) to identify clinical and psychosocial factors related to mandibular-related disability, with the goal of informing future research directions and contributing to the development of more personalized and effective therapeutic strategies in clinical practice.

## 2. Materials and methods

### 2.1 Study design

This cross-sectional study compared individuals diagnosed with TMD to healthy controls. The study was conducted in accordance with the Strengthening the Reporting of Observational Studies in Epidemiology (STROBE) international guidelines, and the A Guideline for Reporting Mediation Analyses of Randomized Trials and Observational Studies (AGReMA) statement (**Supplementary material 1**) [15, 16]. The study was conducted at the Hospital of Guadalajara (Guadalajara, Spain) and a private physical therapy center in Alcalá de Henares (Madrid, Spain), between April 2022 and June 2023.

The study was approved by the Ethics Committee of Universidad de Alcalá (CEID/2021/4/087) and was conducted according to the Declaration of Helsinki. Before any measurement, participants were provided with detailed explanations about all study procedures and signed an informed consent.

### 2.2 Participants

The study included patients with TMD, and patients without pain or disorder related to the temporomandibular region. They were recruited through local advertisements in Alcalá de Henares and Guadalajara in Spain.

### 2.3 Sample size

The sample size calculation was conducted using the R software, for each of the two main objectives of the study.

First, for the objective of evaluating differences in pressure pain threshold (PPT) measurements, the sample size was based on the precision of the adjusted mean difference of a multivariable linear regression model, including age, sex, weight, and height as covariates, using the “MBESS” package, following the proposal of Kelley & Maxwell [17]. The standard deviation of the different PPT measurements was estimated to be 1 kg/cm^2^, based on previously published literature. It was assumed a value of *R*^2^ = 0.4 for the multivariable regression model predicting PPT, and a variance inflation factor for the TMD categorical predictor of 1.25. The width of the 95% confidence interval (CI) assumed to be acceptable was 0.8 kg/cm^2^, with an 80% power, and using Bonferroni’s correction for 4 multiple comparisons. With an expected 10% drop-out rate, the final sample was composed of 164 subjects (82 subjects per group).

For the second objective, evaluating the mediator effect of PPT measurements on active mouth opening differences between TMD and healthy subjects, the sample size was calculated based on the desirable power to detect a significant indirect effect using a percentile bootstrap procedure, following the proposal of Fritz and MacKinnon [18]. The standardized regression coefficient for both paths in the mediation model, namely “a_1_”, “a_2_”, “a_3_”, “a_4_” and “b_1_”, “b_2_”, “b_3_” and “b_4_”, in the conceptual graph (Fig. 1) was assumed to be 0.26 (medium effect size). The final sample, assuming a 10% drop-out rate, was 180 subjects.

**Fig. 1.** Conceptual graph for multiple parallel mediation model. This figure presents the assumed causal associations between temporomandibular joint disorder, mediators and confounding factors and the outcome active mouth opening range of motion. Abbreviations: TMJ: temporomandibular joint; PPT: pressure pain threshold.

### 2.4 Inclusion and exclusion criteria

#### 2.4.1 Temporomandibular disorder subjects

To be eligible for the TMD group, participants had to meet the following criteria: (1) to present with unilateral or bilateral orofacial pain, lasting at least one month; (2) age equal or greater than 18 years; (3) to be diagnosed with TMD according to the classification criteria of diagnostic criteria (DC/TMD) into the category pain or muscular disorders (DC/TMD I). The diagnostic criteria used for determining this TMD subtype were a compound of a complete patient history, including symptoms such as unilateral or bilateral orofacial pain in the masticatory region lasting at least one month, as well as a physical examination which includes palpation of masticatory muscles and temporomandibular joint (TMJ) tissues, range of oral motion and joint sounds examinations. Positive findings about palpation in masticatory muscles, and ruling out tenderness at joint tissues, or joint sounds, confirm a muscle-related disorder according to DC/TMD classification [1].

The following exclusion criteria were considered for TMD patients: (1) previous cervical or cranioencephalic trauma, such as neck whiplash; (2) to have been diagnosed with any systemic disease, such as fibromyalgia or rheumatoid arthritis; (3) any vascular or metabolic concomitant disease; (4) previous surgeries at the cervical or temporomandibular regions; (5) any dental, medical (except casual drugs), or physical therapy treatment within the last 3 months for the temporomandibular or neck regions.

#### 2.4.2 Healthy subjects

The following inclusion criteria were considered for the healthy subjects: (1) Age equal or greater than 18 years; (2) not to have any pain in the temporomandibular region, temporomandibular joint, or cervical region within the last month.

Furthermore, subjects should not present any of the following exclusion criteria: (1) previous cervical or cranioencephalic trauma; (2) to have been diagnosed of any systemic disease, such as fibromyalgia or rheumatoid arthritis; (3) any vascular or metabolic concomitant disease; (4) previous surgeries at the cervical or temporomandibular region.

### 2.5 Variables

All the measurements were conducted at the Hospital of Guadalajara (Guadalajara, Spain), and a private physical therapy clinic in Alcalá de Henares (Madrid, Spain) by three physical therapists with more than 5 years of experience. The evaluators were not blinded to the presence of TMD due to ethical issues within the clinical settings. Demographical data of age, sex, weight and height were collected. All participants were asked not to take analgesic drugs 24 hours before the study measurements.

#### 2.5.1 Pain intensity

Pain intensity within the last week was assessed with a visual analogue scale (VAS) [19]. The VAS comprises a 10-mm horizontal line ranging from 0 (no pain) to 100-mm (“worst imaginable pain”).

#### 2.5.2 Mechanosensitivity

Tissue mechanosensitivity was measured with PPT. The PPT were recorded with a hand-held analog algometer (FPK 20, Wagner Instruments, Greenwich, CT, USA) equipped with a 1-cm^2^ probe, which has demonstrated good reliability in previous studies [14]. Pressure was applied perpendicularly to the surface of each measurement site at an approximate rate of 1 kg/cm^2^ per second. Three consecutive measurements were taken at each site, with a 15-second rest interval between trials. The measurement locations included: masseter muscle, temporalis muscle, lateral pole of the temporomandibular joint (TMJ), and the mandibular branch (V3) of the trigeminal nerve. In TMD participants with bilateral symptoms, the mean of both sides was used; in healthy subjects, the side of measurement was randomly selected.

All measurements were performed with the participant seated in an upright position. For the masseter and temporalis muscles, the most tender point identified during palpation was used as the measurement site. The V3 measurement was taken approximately 2 cm below the corner of the lip, corresponding to the projection of the mental nerve [20]. The patients were instructed to indicate the moment when the sensation changed from pressure to pain.

#### 2.5.3 Range of motion

Mouth opening range of motion was measured actively and passively. The measurements were obtained with the patient seated in an upright position, using a calliper to measure the inter-incisal distance (millimeters). For the active mouth opening, patients were asked to actively open his/her mouth as much as they could, regardless of the presence of pain. For the passive measurement, the evaluator opened the patients’ mouth until he/she noted an end-feel sensation, or the patient reported pain/discomfort with his/her hand to the evaluator.

#### 2.5.4 Health-related quality of life

Health-related quality of life was measured with the Spanish version of the Short-Form 12 survey (SF-12) [21]. This questionnaire is composed of 12 items from the SF-36 extended version. This version was transculturally adapted in the original manuscript including nine European countries, conducted by the International Quality of Life Assessment (IQOLA) Project, showing similar psychometric properties to the SF-36 version and to the versions from other countries [21]. The questionnaire was explained to the participants by a physical therapist who resolved any concerns about the items.

#### 2.5.5 Disability

Craniofacial disability was assessed with the Craniofacial Pain and Disability Inventory (CF-PDI), which was originally developed in Spanish in 2014 [22], Neck-related disability was measured using the Neck Disability Index (NDI), which was transculturally adapted to Spanish in 2008 [23].

#### 2.5.6 Anxiety

The level of anxiety was quantified with the State-Trait Anxiety Inventory (STAI), which was adapted into Spanish in 2014 [24], and has demonstrated good psychometric properties. This questionnaire is composed of two subscales, the State Anxiety Scale (S-Anxiety), which evaluates the current state of anxiety, and the Trait Anxiety Scale (S-Trait), that evaluates general state related to anxiety [24].

### 2.6 Statistical analysis

All analyses were conducted using software R v.4.1.0 (R Core Team (2021). R: A language and environment for statistical computing. R Foundation for Statistical Computing, Vienna, Austria). An alpha value of 0.05 was assumed for all analyses, with 95% confidence intervals (CI).

For the descriptive analysis of quantitative variables, the mean, standard deviation (SD), first and third quartiles, median and range were reported. For categorical variables the absolute frequencies and percentages were reported. Data distribution of quantitative variables was analyzed with histograms, and Q-Q plots. Finally, a correlation matrix between all quantitative variables was also constructed for descriptive purposes. No missing data was present.

Between-group comparisons of outcome measures were analyzed using an ordinary least squares regression model using the R package “rms” (Frank E Harrell Jr, 2022), including age, height, weight and sex as covariates, and the presence of TMD as the predictor of interest. Multiple families were set *a priori* to control for familywise error rate using Bonferroni’s correction: pressure pain threshold (four outcomes), mouth opening (two outcomes), SF-12 questionnaire (2 outcomes) and STAI questionnaire (2 outcomes). The Bonferroni’s correction was applied separately within each family. Finally, residual plots were constructed for each model to evaluate their adequacy.

A multiple parallel mediation analysis was conducted to evaluate whether changes in active mouth opening between healthy controls and individuals with TMD were produced by changes in PPT. The hypothesis set *a priori* was that the presence of TMD would produce a decrease in PPT, which would subsequently lead to a decrease in active mouth opening. For the analysis, age, height, weight and sex were included as covariates, and the four PPT measures as mediator variables. The conceptual graph of the model is presented in Fig. 1. The indirect effect of interest was the combined effect of all four PPT measures, as it was expected that they would be in the same abovementioned direction. The analysis was conducted using the PROCESS function for R, created by Andrew F. Hayes [18]. A percentile bootstrap procedure with 5000 samples was conducted to calculate 95% CI for indirect effects. This statistical method for causal pathways was selected because it evaluates directly the mediation effect, unlike other methods such as the “causal steps approach” of Baron & Kenny, and because it does not assume a distribution for the indirect effect, like does the Sobel method (*i.e.*, assuming normal distribution), which may not hold in real practice [18].

Finally, for the secondary aim of the study regarding the assessment of the relationship between PPT, mental health, physical health and neck disability, with mandibular-related disability (CF-PDI) within subjects with TMD, a beta-regression model was implemented [25]. The selection of a beta-regression model was because the CF-PDI is bounded at the top and bottom, so this model is more appropriate than an ordinary least squares one. The analysis was conducted using the “betareg” package in R [26]. First, since we had many potential predictors for the small available sample size (15 predictors for 85 subjects with TMD), we employed a data reduction procedure which is detailed in **Supplementary material 2**. The regression coefficients of the final model were reported with 95% CI (no multiple comparison correction was performed, because of the exploratory nature of this analysis), as well as diagnostic plots to evaluate the adequacy of the model. The coefficients were also transformed into the odds ratio for the outcome measure as per unit change in the predictor variable, with a value of 1 meaning no association, a value >1 meaning a positive association, and a value <1 meaning a negative association.

## 3. Results

The final sample consisted of 179 participants, including 94 healthy controls (38 female), and 85 individuals with TMD (66 female). Summary descriptive statistics are presented in Table 1, while full descriptive statistics are presented in **Supplementary material 3**. The correlation matrix and histograms for both the TMD and control groups are provided in **Supplementary materials 4 and 5**, respectively.

**Table 1.** Characteristics of included subjects.

Variable		Mean	SD	Median	Mean	SD	Median	Test
Group		No pain (n = 94)			TMJ disorder (n = 85)			
Age, yr		35.415	11.655	31	36.165	12.904	30	*F* = 0.167
Weight, kg		73.379	11.897	74	65.640	13.164	63	*F* = 17.068***
Height, cm		170.755	8.851	172	166.235	8.478	165	*F* = 12.116***
Sex								
	Female	38 (40.4%)			66 (77.6%)			χ^2^ = 23.898***
	Male	56 (59.6%)			19 (22.4%)			
NPRS (rest)					3.251	2.018	3	
NPRS (movement)					4.518	1.875	4	
CF-PDI					32.933	10.049	33	
NDI					15.694	15.094	14	
Headache								
	NO	81 (86.2%)			57 (67.1%)			χ^2^ = 8.182***
	YES	13 (13.8%)			28 (32.9%)			
Previous neck pain								
	NO	72 (76.6%)			52 (61.2%)			χ^2^ = 4.288**
	YES	22 (23.4%)			33 (38.8%)			
Bruxism								
	NO	75 (79.8%)			50 (58.8%)			χ^2^ = 8.343***
	YES	19 (20.2%)			35 (41.2%)			
Dental treatment								
	NO	65 (69.1%)			39 (45.9%)			χ^2^ = 8.993***
	YES	28 (30.9%)			45 (54.1%)			

**p < 0.01, ***p < 0.001. Abbreviations: SD: standard deviation; NPRS: numeric pain rating scale; CF-PDI: Craniofacial Pain and Disability Inventory; NDI: Neck Disability Index.

### 3.1 Between-group differences in outcome measures

The regression models including age, sex, height, and weight as covariates revelated that the TMD group had less range of mouth opening, both active (−6.52 mm; 95% CI, −9.57 to −3.46) and passive (−6.86 mm; 95% CI, −9.72 to −3.99) (Table 2).

**Table 2.** Adjusted between-group differences.

Outcome		No pain (n = 94) Mean (SD)	TMJ Disorder (n = 85) Mean (SD)	Adjusted between-group mean difference*, 95% CI
Mouth opening (active), mm		41.70 (7.34)	34.24 (9.48)	−6.52 (−9.57 to −3.46)
Mouth opening (passive), mm		46.58 (6.77)	39.90 (9.65)	−6.86 (−9.72 to −3.99)
Pressure pain threshold, kg/m^2^				
	Masseter	1.89 (0.61)	1.24 (0.55)	−0.45 (−0.67 to −0.23)
	Temporalis	2.96 (1.26)	2.02 (0.85)	−0.56 (−0.95 to −0.16)
	Lateral TMJ	2.16 (0.69)	1.22 (0.52)	−0.76 (−1.00 to −0.52)
	V3	1.75 (0.66)	1.35 (0.71)	−0.22 (−0.49 to 0.05)
Short-Form 12				
	Mental	50.43 (8.52)	49.24 (8.50)	0.20 (−2.95 to 3.35)
	Physical	54.01 (5.19)	50.70 (8.09)	−2.84 (−5.30 to −0.39)
State-Trait Anxiety Inventory				
	State	20.64 (11.11)	27.70 (11.92)	4.75 (0.58 to 8.91)
	Trait	22.53 (12.35)	29.76 (10.07)	3.34 (−0.47 to 7.15)

*Differences adjusted for age, height, weight and sex. Abbreviations: SD: standard deviation; 95% CI: 95% confidence interval; TMJ: temporomandibular joint; V3: mandibular division of trigeminal nerve. Confidence intervals are adjusted using Bonferroni’s correction.

Regarding tissue mechanosensitivity, the TMD group showed less PPT in masseter muscle (−0.45 kg/cm^2^; 95% CI, −0.67 to −0.23), temporalis (−0.56 kg/cm^2^; 95% CI, −0.95 to −0.16) and lateral TMJ (−0.76 kg/cm^2^; 95% CI, −1.00 to −0.52), but not V3 point (Table 2)

Finally, the TMD group reported lower scores on the physical component of the SF-12 questionnaire (−2.84; 95% CI, −5.30 to −0.39), and higher scores on the State Anxiety subscale of the STAI (4.75; 95% CI, 0.58 to 8.91) (Table 2).

The residual plots of the regression models are presented in **Supplementary material 6**.

### 3.2 Mediation analysis for changes in active mouth opening

The multiple parallel mediation model revealed that the reduction in active mouth opening observed in individuals with TMD was not significantly mediated by a decreased PPT values. While a significant direct effect of TMD on active mouth opening was identified (−7.50 mm; 95% CI, −10.59 to −4.40), the total indirect effect through the four PPT measures was not statistically significant (0.98 mm; 95% CI, −0.87 to 3.12) (Table 3). The full statistics for mediation analysis are presented in **Supplementary material 7**.

**Table 3.** Results of multiple parallel mediation model for active mouth opening.

Variable		Effect	Standard error	95% CI*
Direct effect		−7.50	1.57	−10.59 to −4.40
Indirect effects				
	Total	0.98	1.01	−0.87 to 3.12
	PPT (masseter)	−0.19	0.87	−1.86 to 1.56
	PPT (temporalis)	0.39	0.53	−0.46 to 1.69
	PPT (lateral TMJ)	−0.95	0.50	−2.07 to −0.12
	PPT (V3)	1.72	1.15	−0.44 to 4.05

In the model, active mouth opening is the dependent variable, the presence of temporomandibular joint disorder is the predictor variable, and the pressure pain threshold measures are the mediator variables. Age, weight, height and sex were included as covariates. *95% confidence intervals using a percentile bootstrap with 5000 samples. Abbreviations: CI: confidence interval; PPT: pressure pain threshold; TMJ: temporomandibular joint; V3: mandibular division of trigeminal nerve.

### 3.3 Relationship between outcome measures and mandibular-related disability

The backward deletion procedure based on variable clustering revealed that the final reduced model, without PPT and mental health outcomes, performed comparably to the full model (**Supplementary material 8**). This suggests that neither mechanosensitivity nor mental health measures were significantly associated with craniofacial disability as measured by the Craniofacial Pain and Disability Inventory (CF-PDI).

Diagnostic plots of the final model are presented in **Supplementary material 8**. One participant (ID = 36) was excluded from the final analysis based on model diagnostics. After adjusting for age, sex, weight and height, neither the NDI nor the SF-12 physical subscale showed a significant relationship with CF-PDI (Odds Ratio (OR) = 1.004 per unit change in NDI; and OR = 0.99, per unit change in SF-12 physical) (Table 4).

**Table 4.** Coefficients for predictors included in the final beta-regression model for predicting Craniofacial Pain and Disability Inventory (n = 84).

Parameter	Estimate	Standard error	95% CI for estimate	OR
Intercept	−0.560	0.450	−1.440 to 0.322	-
Age	0.001	0.004	−0.006 to 0.008	1.001
Sex (male)	−0.210	0.140	−0.481 to 0.062	0.810
Weight	0.004	0.004	−0.005 to 0.012	1.004
Height	−0.001	0.001	−0.002 to 0.001	0.999
SF-12 (physical)	−0.007	0.006	−0.018 to 0.004	0.993
NDI	0.004	0.004	−0.002 to 0.100	1.004
Standardized weighted residuals 2:				
Min	1Q	Median	3Q	Max
−2.7217	−0.7606	0.2624	0.7418	1.9387
Phi coefficients (precision model with identity link):				
	Estimate	Std. Error	*z* value	*p*-value
(phi)	26.863	4.078	6.588	4.47 × 10^−11^
Type of estimator: ML (maximum likelihood)				
Log-likelihood: 84.57 on 8 *Df*				
Pseudo *R*-squared: 0.08659				
Number of iterations: 15 (BFGS) + 2 (Fisher scoring)				

*Abbreviations: CI: confidence interval; OR: odds ratio; SF-12: Short-Form 12; NDI: Neck Disability Index; Min: minimum value; Max: maximum value; Std. Error: standard error; Df: degrees of freedom; BFGS: Broyden-Fletcher-Goldfarb-Shanno.*

## 4. Discussion

This cross-sectional study included a total of 179 participants (85 with TMD). Within the TMD group, 66 participants (77.65%) were women, which aligns with previous research indicating a higher prevalence of TMD among females [27].

The results of this study revealed that individuals with TMD exhibited significantly reduced active and passive mouth opening, increased tissue mechanosensitivity (as indicated by lower PPT values) in the masseter and temporalis muscles and the lateral TMJ, lower scores on the physical component of the SF-12 questionnaire, and higher levels of state anxiety as measured by the STAI (Table 2).

Consistent with earlier studies, our results confirmed a reduction in both active and passive mouth opening in individuals with TMD compared to healthy controls [9].

Several previous studies have investigated alterations in tissue mechanosensitivity in patients with TMD, examining both local and remote anatomical sites [28]. Most of these studies have focused on PPT assessments at the masseter and temporalis muscles, as well as the TMJ [28]. However, to our knowledge, only one published study has evaluated PPT at the peripheral branches of the trigeminal nerve [29].

Our findings are consistent with prior studies reporting reduced PPT values at the local muscles and TMJ sites [28]. However, our results differ from those of Fernández-de-las-Peñas *et al*. [29], who found a significant bilateral decrease in PPT at the supra-orbital (V1), infra-orbital (V2), and mental (V3) branches of the trigeminal nerve in TMD patients [30]. However, in our study, no significant reduction in PPT was observed at the V3 measurement site (mean difference, −0.22; 95% CI, −0.49 to 0.05). These discrepancies may be due to the fact that we controlled for demographical confounding factors (age, sex, height and weight), while the study of Fernández-de-las-Peñas *et al*. [29] did not adequately adjust for these factors.

Finally, regarding psychosocial variables, there are mixed findings within existing literature [30, 31, 32]. While some studies have reported associations between TMD and psychological variables such as depression symptoms and somatization [32, 33], others have found no significant relationship with anxiety symptoms measured using the STAI questionnaire [30, 31].

In our study, there was a statistically significant increase in scores on the state subscale of the STAI among participants with TMD (mean difference, 4.75; 95% CI, 0.58 to 8.91), but not on the trait subscale. The fact that this difference was small, and only significant in the state but not the trait subscale, limits the clinical relevance of these findings, especially in light of previous studies that have not reported significant differences in anxiety between TMD and control groups [30, 31, 34].

Similarly, it was only found a small but statistically significant reduction in the SF-12 physical subscale among TMD patients (mean difference; −2.84; 95% CI, −5.30 to −0.39), with no corresponding difference observed in the mental subscale. These results are in agreement with previous studies that have found a modest association between TMD and the physical, but not mental, component of the SF-12 [35]. Furthermore, other studies have found no correlation between SF-12 subscales and the Fonseca Anamnestic Index questionnaire [36]. Overall, there is a lack of evidence for stablishing an association between TMD and the STAI and SF-12 questionnaires in young adults.

One of the main goals of the treatment for patients with TMD is to restore mouth opening range of motion [3]. Despite the extensive literature showing a decrease in the range of mouth opening in patients with TMD [3, 5], the causal mechanisms for its reduction are not clear yet.

Two main hypotheses have been proposed to explain the reduction in mouth opening range of motion observed in individuals with TMD. The first involves alterations in temporomandibular disc kinematics which may act as a mechanical “block” to movement. However, such disc displacement or dysfunction is not consistently observed across all TMD patients, and can also be present in asymptomatic individuals, suggesting that it may not fully account for the observed limitations in jaw mobility [10].

The second hypothesis, which was directly evaluated in the present study, posits that increased local tissue mechanosensitivity, specially within the masticatory muscles [13], leads to a reduction in mouth opening range of motion in an attempt to avoid the reproduction of pain due to the stress imposed during this movement over the sensitized tissues [13].

In the present study, a reduction was observed in active and passive mouth opening, as well as lower PPT values over the masseter and temporalis muscles, and lateral TMJ in individuals with TMD. However, the multiple parallel mediation analysis revealed that the reduction of active mouth opening because of the presence of TMD, was not mediated by changes in local tissue mechanosensitivity (total indirect effect, 0.98; 95% CI, −0.87 to 3.12) (Table 3).

To date, there is limited literature applying analysis in TMD populations [37, 38], and to our knowledge, no published studies evaluated the causal mechanisms underlying the reduction of mouth opening in this population.

Our mediation model assumed a linear relationship between tissue mechanosensitivity and active mouth opening. This relationship could also be modeled as non-linear, for example using fractional polynomials, which could yield different results [39]. Furthermore, it is also possible that one or both paths of the indirect effects of the causal mediation model are moderated by one or more variables, leading to a moderated-mediated model (*i.e.*, the indirect effect can be present for some subgroup of subjects with TMD but not for others), or even more complex causal structures [18].

Both non-linearity [39] and complex causal models require a large sample size in order to adequately estimate the desired parameters [18]. For this reason, it was decided to test the model using linearity assumption and no moderated-mediated effects. Therefore, although these represent preliminary results, future studies should evaluate more complex mediated models in order to evaluate other possible paths of causality between the presence of TMD, the alteration of tissue mechanosensitivity and the reduction of active mouth opening.

Regarding the analysis of the relationship between outcome measures and mandibular-related disability, measured with the CF-PDI, no significant associations were observed in our study (Table 4). Previous studies have evaluated the relationship between mandibular-related disability and PPT measures [40], and the NDI [6] in subjects with TMD. Silveira *et al*. [40] showed moderate negative correlations between PPT measured at the temporalis, masseter, sternocleidomastoid and upper trapezius muscles, and the Limitations of Daily Functions in TMD questionnaire.

The discrepancies between their findings, and the lack of association observed in our study, may be explained by several factors. Notably, Silveira *et al*. [40] conducted their analysis on a mixed sample composed of 20 individuals with chronic TMD and 20 healthy controls. If there are differences between TMD and healthy controls in both variables (PPT and mandibular-related disability), this would induce a spurious association between them, even if there is no relationship at all within TMD patients. Furthermore, they did not control for demographical confounding variables [40]. Both factors can dramatically affect the estimation of associations between variables, serving as an explanation for the discrepancies between their results and the ones obtained in the current study.

In 2020, Cuenca-Martínez *et al*. [6] meta-analyzed data regarding the bivariate association between mandibular-related disability and the NDI. Based on 6 studies, they obtained a pooled strong association between them (*r* = 0.72; 95% CI, 0.56 to 0.82) [6]. However, none of their six included studies controlled for demographical confounding factors, and they also included the study of Silveira *et al*. [40] which has the abovementioned limitations.

Overall, these statistical limitations could have led to an overestimation of the relationship between mandibular-related and neck-related disability in patients with TMD. Based on these limitations, and the results of our current study, it is possible that the true association between mandibular-related disability and the Neck Disability Index (NDI) is smaller than previously assumed. Future studies should consider using multivariable regression models with the aim of controlling for confounding variables, as well as to use the beta-regression model to take into account the bounded nature of disability questionnaires, which should not be modeled with the classical linear regression model [26, 41].

The present study has several limitations that should be acknowledged. First, due to its cross-sectional design, only associations and not causal inference can be established, even when mediation analyses are employed. Future studies using longitudinal cohort designs are necessary to validate potential causal pathways.

Second, due to the non-probabilistic sampling method employed, there is a possibility for selection bias. More research with this same methodology in other populations should be conducted to confirm the generalizability of our findings.

Third, future studies should consider confounding factors when evaluating the association between outcomes in subjects with TMD.

Furthermore, researchers should use adequate multivariable regression models (such as the beta-regression model), in an attempt to consider the bounded and non-linear nature of some outcome measures (*e.g.*, CF-PDI and NDI). Also, future studies should implement moderated-mediation models, including non-linear associations and other possible mediators, to lead some light into the causal pathways in subject with TMD.

Finally, results of the present study could serve as a first step into discerning the causal pathways that lead TMD to a reduction in active mouth opening. Future research should consider the addition of other mediators to the models, the presence of non-linear causal paths, and the possibility of mediated-moderated effects.

## 5. Conclusions

This cross-sectional study showed that individuals with TMD exhibit decreased mouth opening range of motion, and increased mechanosensitivity of masticatory muscles, compared to healthy controls. Furthermore, tissue mechanosensitivity does not mediate the reduction in active mouth opening observed in TMD. Additionally, no significant associations were found between PPT measures, mental health outcomes, NDI, and mandibular-related disability. These results highlight the complexity of TMD and the need for further research to better understand the mechanisms underlying functional limitations in this population.

## Supplementary Material

Supplementary material associated with this article can be found, in the online version, at https://files.jofph.com/ files/article/1966370245760172032/attachment/ Supplementary%20material.docx.


