# Supplementary material for: Regional cutaneous and muscle sensitivity does not mediate changes in active mouth opening in temporomandibular disorders: a cross-sectional study
Source: J Oral Facial Pain Headache. 2025 Sep 12;39(3):95–104. doi: 10.22514/jofph.2025.051 (PMC12520430; doi:10.22514/jofph.2025.051)
Supplement: Supplementary file 1 [file Supplementary-material.docx]

Supplementary material

# Supplementary material 1. STROBE and AGReMA checklist.

Supplementary Table 1. STROBE Statement—checklist of items that should be included in reports of observational studies.

|  | Item No. | Recommendation | Page No. |
| --- | --- | --- | --- |
| Title and abstract | 1 | (a) Indicate the study’s design with a commonly used term in the title or the abstract | 1 |
|  |  | (b) Provide in the abstract an informative and balanced summary of what was done and what was found | 2 |
| Introduction | | | |
| Background/rationale | 2 | Explain the scientific background and rationale for the investigation being reported | 3–4 |
| Objectives | 3 | State specific objectives, including any prespecified hypotheses | 3–4 |
| Methods | | | |
| Study design | 4 | Present key elements of study design early in the paper | 5 |
| Setting | 5 | Describe the setting, locations, and relevant dates, including periods of recruitment, exposure, follow-up and data collection | 5 |
| Participants | 6 | (a) Cohort study—Give the eligibility criteria, and the sources and methods of selection of participants. Describe methods of follow-up  Case-control study—Give the eligibility criteria, and the sources and methods of case ascertainment and control selection. Give the rationale for the choice of cases and controls  Cross-sectional study—Give the eligibility criteria, and the sources and methods of selection of participants | 6 |
|  |  | (b) Cohort study—For matched studies, give matching criteria and number of exposed and unexposed  Case-control study—For matched studies, give matching criteria and the number of controls per case |  |
| Variables | 7 | Clearly define all outcomes, exposures, predictors, potential confounders and effect modifiers. Give diagnostic criteria, if applicable | 6–8 |
| Data sources/measurement | 8* | For each variable of interest, give sources of data and details of methods of assessment (measurement). Describe comparability of assessment methods if there is more than one group | 6–8 |
| Bias | 9 | Describe any efforts to address potential sources of bias | 6 |
| Study size | 10 | Explain how the study size was arrived at | 5–6 |
| Quantitative variables | 11 | Explain how quantitative variables were handled in the analyses. If applicable, describe which groupings were chosen and why | 8–9 |
| Statistical methods | 12 | (a) Describe all statistical methods, including those used to control for confounding | 8–9 |
|  |  | (b) Describe any methods used to examine subgroups and interactions | 8–9 |
|  |  | (c) Explain how missing data were addressed | NA |
|  |  | (d) Cohort study—If applicable, explain how loss to follow-up was addressed  Case-control study—If applicable, explain how matching of cases and controls was addressed  Cross-sectional study—If applicable, describe analytical methods taking account of sampling strategy | NA |
|  |  | (e) Describe any sensitivity analyses | NA |
| Results | | | |
| N | 13* | (a) Report numbers of individuals at each stage of study—*e.g.*, numbers potentially eligible, examined for eligibility, confirmed eligible, included in the study, completing follow-up and analysed | 10 |
|  |  | (b) Give reasons for non-participation at each stage | NA |
|  |  | (c) Consider use of a flow diagram | 10 |
| Descriptive data | 14* | (a) Give characteristics of study participants (*e.g.*, demographic, clinical, social) and information on exposures and potential confounders | 10 |
|  |  | (b) Indicate number of participants with missing data for each variable of interest | NA |
|  |  | (c) Cohort study—Summarise follow-up time (*e.g.*, average and total amount) |  |
| Outcome data | 15* | Cohort study—Report numbers of outcome events or summary measures over time |  |
|  |  | Case-control study—Report numbers in each exposure category, or summary measures of exposure |  |
|  |  | Cross-sectional study—Report numbers of outcome events or summary measures | 10 |
| Main results | 16 | (a) Give unadjusted estimates and, if applicable, confounder-adjusted estimates and their precision (*e.g.*, 95% confidence interval). Make clear which confounders were adjusted for and why they were included | 10 |
|  |  | (b) Report category boundaries when continuous variables were categorized | NA |
|  |  | (c) If relevant, consider translating estimates of relative risk into absolute risk for a meaningful time period | NA |
| Other analyses | 17 | Report other analyses done—*e.g.*, analyses of subgroups and interactions, and sensitivity analyses | NA |
| Discussion | | | |
| Key results | 18 | Summarise key results with reference to study objectives | 11 |
| Limitations | 19 | Discuss limitations of the study, taking into account sources of potential bias or imprecision. Discuss both direction and magnitude of any potential bias | 15 |
| Interpretation | 20 | Give a cautious overall interpretation of results considering objectives, limitations, multiplicity of analyses, results from similar studies and other relevant evidence | 15 |
| Generalisability | 21 | Discuss the generalisability (external validity) of the study results | 15 |
| Other information | | | |
| Funding | 22 | Give the source of funding and the role of the funders for the present study and, if applicable, for the original study on which the present article is based | 17 |

*Give information separately for cases and controls in case-control studies and, if applicable, for exposed and unexposed groups in cohort and cross-sectional studies.

Note: An Explanation and Elaboration article discusses each checklist item and gives methodological background and published examples of transparent reporting. The STROBE checklist is best used in conjunction with this article (freely available on the Web sites of PLOS Medicine at <http://www.plosmedicine.org/>, Annals of Internal Medicine at <http://www.annals.org/>, and Epidemiology at <http://www.epidem.com/>). Information on the STROBE Initiative is available at [www.strobe-statement.org](https://www.strobe-statement.org/).

Supplementary Table 2. AGReMA checklist.

| Section/Topic | Item Number | Item Description | Reported on page No |
| --- | --- | --- | --- |
| Title and abstract | | | |
| Title | 1 | Identify that the study uses mediation analysis | 1 |
| Abstract | 2 | Provide a structured summary of the objectives, methods, results, and conclusions specific to mediation analyses | 5 |
| Introduction | | | |
| Background and rationale | 3 | Describe the study background and theoretical rationale for investigating the mechanisms of interest. Include supporting evidence or theoretical rationale for why the intervention or exposure might have a causal relationship with the proposed mediators. Include supporting evidence or theoretical rationale for why the mediators might have a causal relationship with the outcomes | 7 |
| Objectives | 4 | State the objectives of the study specific to the mechanisms of interest. The objectives should specify whether the study aims to test or estimate the mechanistic effects | 8 |
| Methods | | | |
| Study registration | 5 | If applicable, provide references to any protocols or study registrations specific to the mediation analysis, and highlight any deviations from the planned protocol | NA |
| Study design and source of data | 6 | Specify the design of the original study that was used in mediation analyses and where the details can be accessed, supported by a reference. If applicable, describe study design features that are relevant to mediation analyses | NA |
| Participants | 7 | Describe the target population, eligibility criteria specific to mediation analyses, study locations and study dates (start of participant enrolment and end of follow-up) | 10–11 |
| Sample Size | 8 | State whether a sample size calculation was conducted for mediation analyses. If so, explain how it was calculated | 10 |
| Effects of interest | 9 | Specify the effects of interest | 10 |
| Assumed causal model | 10 | Include a graphic representation of the assumed causal model including the exposure, mediator, outcome, and possible confounders | **Supplementary material 2** |
| Causal assumptions | 11 | Specify assumptions about the causal model | 14 |
| Measurement | 12 | Clearly describe the interventions or exposures, mediators, outcomes, confounders, and moderators that were used in the analyses. Specify how and when they were measured, the measurement properties, and whether blinded assessment was used | 10–13 |
| Measurement levels | 13 | If relevant, describe the levels at which the exposure, mediator, and outcome were measured | NA |
| Statistical methods | 14 | Describe the statistical methods used to estimate the causal relationships of interest. This description should specify analytical strategies used to reduce confounding, model building procedures, justification for the inclusion or exclusion of possible interaction terms, modelling assumptions, and methods used to handle missing data. Provide a reference to the statistical software and package used | 14 |
| Sensitivity analyses | 15 | Describe any sensitivity analyses that were used to explore causal or statistical assumptions and the influence of missing data | 14 |
| Ethical approval | 16 | Name the institutional research board or ethics committee that approved the study. Provide a description of participant informed consent or ethics committee waiver of informed consent | 9 |
| Results | | | |
| Participants | 17 | Describe baseline characteristics of participants included in mediation analyses. Report the total sample size and number of participants lost during follow-up or with missing data | 16 |
| Outcomes and estimates | 18 | Report point estimates and uncertainty estimates for the exposure-mediator and mediator-outcome relationships. If inference concerning the causal relationship of interest is considered feasible given the causal assumptions, report the point estimate and uncertainty estimate | 16–17 and **Supplementary material 8** |
| Sensitivity parameters | 19 | Report the results from any sensitivity analyses used to assess robustness of the causal or statistical assumptions, and the influence of missing data | NA |
| Discussion | | | |
| Limitations | 20 | Discuss the limitations of the study including potential sources of bias | 22 |
| Interpretation | 21 | Interpret the estimated effects considering the study’s magnitude and uncertainty, plausibility of the causal assumptions, limitations, generalizability of the findings and results from relevant studies | 20, 22 and 23 |
| Implications | 22 | Discuss the implications of the overall results for clinical practice, policy, and science | 22–23 |
| Other information | | | |
| Funding and role of  sponsor | 23 | List all sources of funding or sponsorship for the mediation analysis and the role of the funders/sponsors in the conduct of the study, writing of the manuscript, and decision to submit for publication. | 1 |
| Conflicts of interest and financial disclosures | 24 | State any conflicts of interest and financial disclosures for all authors | 1 |
| Data and code | 25 | Authors are encouraged to provide a statement for sharing data and code for the mediation analysis | **Supplementary material 8** |

From: Lee H, Cashin AG, Lamb SE, Hopewell S, Vansteelandt S, VanderWeele TJ, *et al*. A Guideline for Reporting Mediation Analyses of Randomized Trials and Observational Studies. The AGReMA Statement. JAMA. 2021;326(11):1045–1056. doi:10.1001/jama.2021.14075

AGReMA is designed for articles that report mediation analyses of randomized trials or observational studies.

For more information, visit: [agrema-statement.org](https://agrema-statement.org/).

# Supplementary material 2. Data reduction procedure for beta regression model.

First, we selected *a priori* clusters of variables based on clinical knowledge, before conducting any data analysis, which were PPT measures, and mental health measures (SF-12 mental subscale, and STAI questionnaire).

Second, a data redundancy analysis was performed to evaluate if any of the predictors of interest (excluding demographic covariables) was redundant, so it would not be included in the beta-regression model, using the function “redun” from the R package “Hmisc” (Frank E Harrell Jr, 2022), with a cut-off point of *R*^2^ of 0.70. None of the predictors seemed to be redundant.

The initial established clusters were formally evaluated afterwards using a variable cluster analysis with all candidate predictors (excluding demographics), using the function “varclus” of the package “Hmisc”, based on the Hoeffding’s D similarity measure. This analysis revealed the same clusters established based on clinical knowledge.

After that, it was analyzed if a single score for each cluster could be used in the final model, based on a sparse principal component analysis, using the function “sPCAgrid” from the “pcaPP” package in R (Peter Filzmoser, Heinrich Fritz and Klaudius Kalcher, 2023). However, the percentage of variance explained within each cluster by the first principal component was very low (25% for PPT cluster and 33.33% for mental health cluster).

Based on these analyses, we finally decided to perform a backward stepwise regression based on cluster priority. In the first model, all predictors were included. In the second model, the PPT cluster variables were excluded. And in the final model, the mental health cluster variables were excluded. Models were compared using a likelihood ratio test and comparing the magnitude of the Akaike Information Criterion (AIC). If the likelihood ratio test was not significant and the change in AIC was not relevant, the reduced model was retained. These analyses were performed without knowledge of the significance of individual regression coefficients within each cluster.

# Supplementary material 3. Full descriptive statistics.

Supplementary Table 3. Full descriptive statistics of no pain group.

| Variable | | N | Mean | SD | Min | Pctl. 25 | Pctl. 50 | Pctl. 75 | Max |
| --- | --- | --- | --- | --- | --- | --- | --- | --- | --- |
| No pain group (n = 94) | | | | | | | | | |
| Age, yr | | 94 | 35.415 | 11.655 | 19 | 26 | 31 | 43.5 | 65 |
| Weight, kg | | 94 | 73.379 | 11.897 | 50 | 64.25 | 74 | 80.75 | 100 |
| Height, cm | | 94 | 170.755 | 8.851 | 150 | 163 | 172 | 178 | 187 |
| Sex | | 94 |  |  |  |  |  |  |  |
|  | Female | 38 | 40.4% |  |  |  |  |  |  |
|  | Male | 56 | 59.6% |  |  |  |  |  |  |
| Pressure pain threshold, kg/cm^2^ | | | | | | | | | |
| Masseter | | 94 | 1.893 | 0.605 | 0.4 | 1.462 | 1.815 | 2.23 | 3.23 |
| Temporalis | | 94 | 2.955 | 1.259 | 0.6 | 2.13 | 2.72 | 3.805 | 5.8 |
| Lateral TMJ | | 94 | 2.158 | 0.693 | 0.9 | 1.7 | 2 | 2.423 | 4.5 |
| V3 | | 94 | 1.749 | 0.662 | 0.45 | 1.308 | 1.73 | 2.122 | 4.37 |
| Mouth opening (passive), mm | | 94 | 46.579 | 6.785 | 28.9 | 42.125 | 46.15 | 50.75 | 64 |
| Mouth opening (active), mm | | 94 | 41.698 | 7.338 | 25.2 | 36.2 | 42 | 46.15 | 59.77 |
| SF-12 (physical) | | 94 | 54.01 | 5.19 | 35.68 | 52.08 | 55.5 | 56.812 | 62.95 |
| SF-12 (mental) | | 94 | 50.432 | 8.524 | 25.73 | 45.392 | 52.89 | 57.14 | 63.07 |
| STAI (state) | | 94 | 20.642 | 11.109 | 0 | 10.418 | 22.335 | 29 | 51.67 |
| STAI (trait) | | 94 | 22.534 | 12.346 | 0 | 13.33 | 23.165 | 32 | 48.33 |
| Neck Disability Index | | 94 | 3.128 | 5.144 | 0 | 0 | 0 | 4 | 20 |
| Headache diagnosed | | 94 |  |  |  |  |  |  |  |
|  | NO | 82 | 87.2% |  |  |  |  |  |  |
|  | YES | 12 | 12.8% |  |  |  |  |  |  |
| Headache (self-reported) | | 94 |  |  |  |  |  |  |  |
|  | NO | 81 | 86.2% |  |  |  |  |  |  |
|  | YES | 13 | 13.8% |  |  |  |  |  |  |
| Previous neck pain | | 94 |  |  |  |  |  |  |  |
|  | NO | 72 | 76.6% |  |  |  |  |  |  |
|  | YES | 22 | 23.4% |  |  |  |  |  |  |
| Bruxism | | 94 |  |  |  |  |  |  |  |
|  | NO | 75 | 79.8% |  |  |  |  |  |  |
|  | YES | 19 | 20.2% |  |  |  |  |  |  |
| Dental treatment | | 94 |  |  |  |  |  |  |  |
|  | NO | 65 | 69.1% |  |  |  |  |  |  |
|  | YES | 29 | 30.9% |  |  |  |  |  |  |

Abbreviations: SD: standard deviation; Pctl.: percentile; TMJ: temporomandibular joint; SF-12: Short-Form 12 questionnaire; STAI: State-Trait Anxiety Inventory; V3: mandibular division of trigeminal nerve.

Supplementary Table 4. Full descriptive statistics of the painful group.

| Variable | | N | Mean | SD | Min | Pctl. 25 | Pctl. 50 | Pctl. 75 | Max |
| --- | --- | --- | --- | --- | --- | --- | --- | --- | --- |
| Temporomandibular Joint Disorder group (n = 85) | | | | | | | | | |
| Age, yr | | 85 | 36.165 | 12.904 | 18 | 26 | 30 | 46 | 62 |
| Weight, kg | | 85 | 65.64 | 13.164 | 47 | 56 | 63 | 74 | 135 |
| Height, cm | | 85 | 166.235 | 8.478 | 149 | 160 | 165 | 172 | 187 |
| Sex | | 85 |  |  |  |  |  |  |  |
|  | Female | 66 | 77.6% |  |  |  |  |  |  |
|  | Male | 19 | 22.4% |  |  |  |  |  |  |
| NPRS (rest) | | 85 | 3.251 | 2.018 | 0 | 1.7 | 3 | 4 | 9.3 |
| NPRS (movement) | | 85 | 4.518 | 1.875 | 0 | 3 | 4 | 6 | 8.8 |
| Pressure pain threshold, kg/cm^2^ | | | | | | | | | |
| Masseter | | 85 | 1.235 | 0.55 | 0.2 | 0.9 | 1.3 | 1.53 | 2.8 |
| Temporalis | | 85 | 2.02 | 0.846 | 0.2 | 1.5 | 2 | 2.5 | 5.3 |
| Lateral TMJ | | 85 | 1.219 | 0.521 | 0.13 | 0.9 | 1.23 | 1.57 | 2.6 |
| V3 | | 85 | 1.347 | 0.712 | 0.2 | 0.86 | 1.23 | 1.67 | 4.03 |
| Mouth opening (passive), mm | | 85 | 39.897 | 9.651 | 10 | 35 | 40.6 | 46 | 69 |
| Mouth opening (active), mm | | 85 | 34.241 | 9.479 | 11 | 28.2 | 36.2 | 39.67 | 64 |
| SF-12 (physical) | | 85 | 50.703 | 8.093 | 24.16 | 45.34 | 53.9 | 56.01 | 61.11 |
| SF-12 (mental) | | 85 | 49.24 | 8.496 | 24.33 | 45.17 | 51.33 | 55.2 | 62.1 |
| CF-PDI | | 85 | 32.933 | 10.049 | 0 | 26.98 | 33.33 | 38.55 | 53.2 |
| STAI (state) | | 85 | 27.698 | 11.917 | 0 | 20 | 28 | 33 | 68.33 |
| STAI (trait) | | 85 | 29.761 | 10.073 | 6.67 | 24 | 31 | 35 | 61.67 |
| Neck Disability Index | | 85 | 15.694 | 15.094 | 0 | 0 | 14 | 24 | 60 |
| Headache diagnosed | | 85 |  |  |  |  |  |  |  |
|  | NO | 66 | 77.6% |  |  |  |  |  |  |
|  | YES | 19 | 22.4% |  |  |  |  |  |  |
| Headache (self-reported) | | 85 |  |  |  |  |  |  |  |
|  | NO | 57 | 67.1% |  |  |  |  |  |  |
|  | YES | 28 | 32.9% |  |  |  |  |  |  |
| Previous neck pain | | 85 |  |  |  |  |  |  |  |
|  | NO | 52 | 61.2% |  |  |  |  |  |  |
|  | YES | 33 | 38.8% |  |  |  |  |  |  |
| Bruxism | | 85 |  |  |  |  |  |  |  |
|  | NO | 50 | 58.8% |  |  |  |  |  |  |
|  | YES | 35 | 41.2% |  |  |  |  |  |  |
| Dental treatment | | 85 |  |  |  |  |  |  |  |
|  | NO | 39 | 45.9% |  |  |  |  |  |  |
|  | YES | 46 | 54.1% |  |  |  |  |  |  |

Abbreviations: SD: standard deviation; Pctl.: percentile; TMJ: temporomandibular joint; SF-12: Short-Form 12 questionnaire; CF-PDI: Craniofacial Pain and Disability Inventory; STAI: State-Trait Anxiety Inventory; V3: mandibular division of trigeminal nerve.

# Supplementary material 4. Correlation matrix and histograms (no pain group).


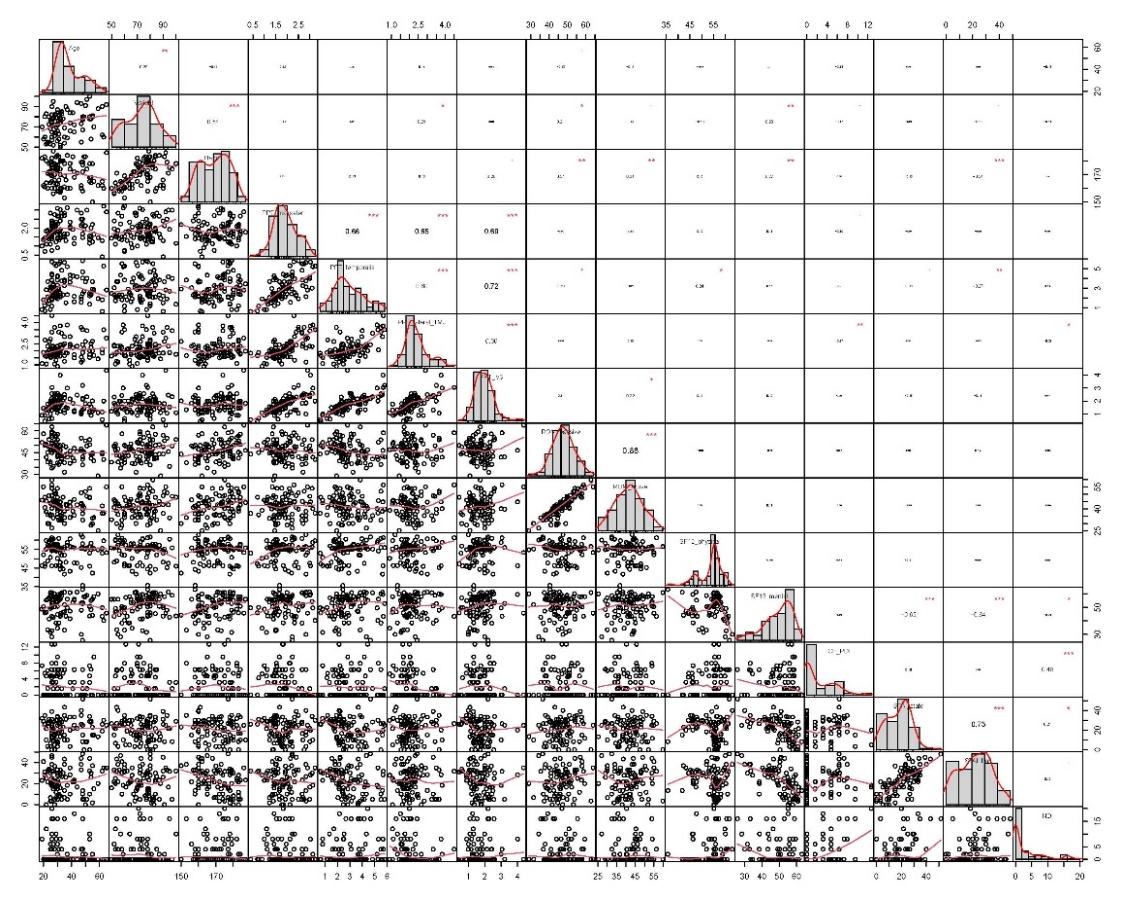


Supplementary Fig. 1. Correlation matrix and histograms (no pain group).

# Supplementary material 5. Correlation matrix and histograms (TMJ disorder group).


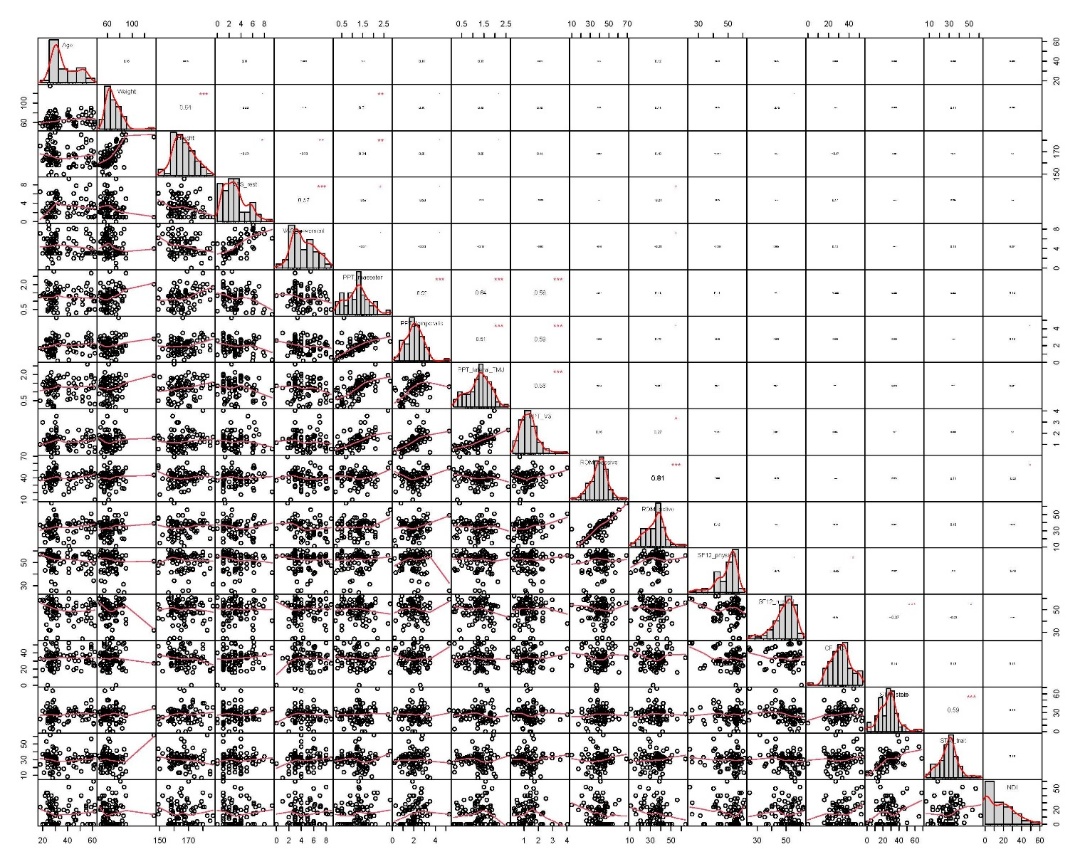


Supplementary Fig. 2. Correlation matrix and histograms (TMJ disorder group).

# Supplementary material 6. Residual plots adjusted-between group differences. Ordinary least squares (OLS) models. Plots are residuals versus fitted values within each group, and Q-Q plots.


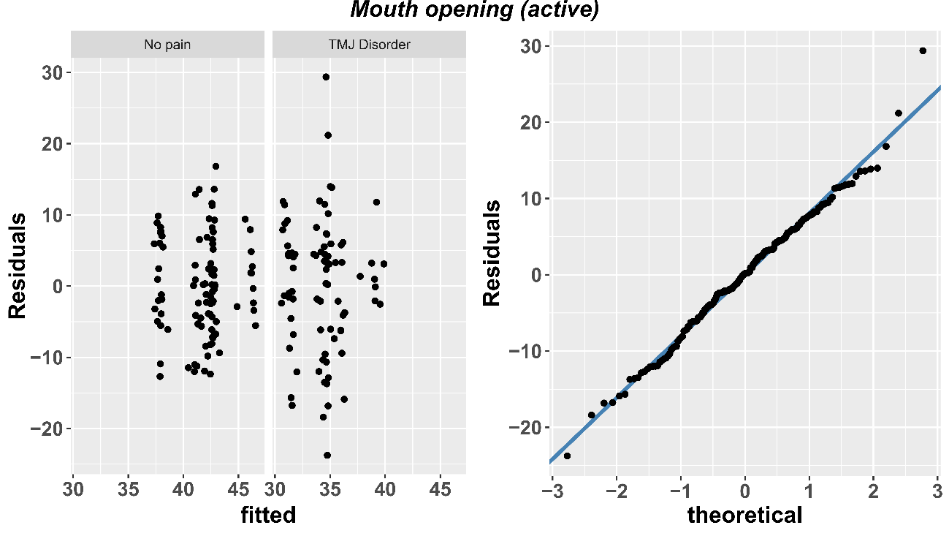


Supplementary Fig. 3. Residual plots for active mouth opening.


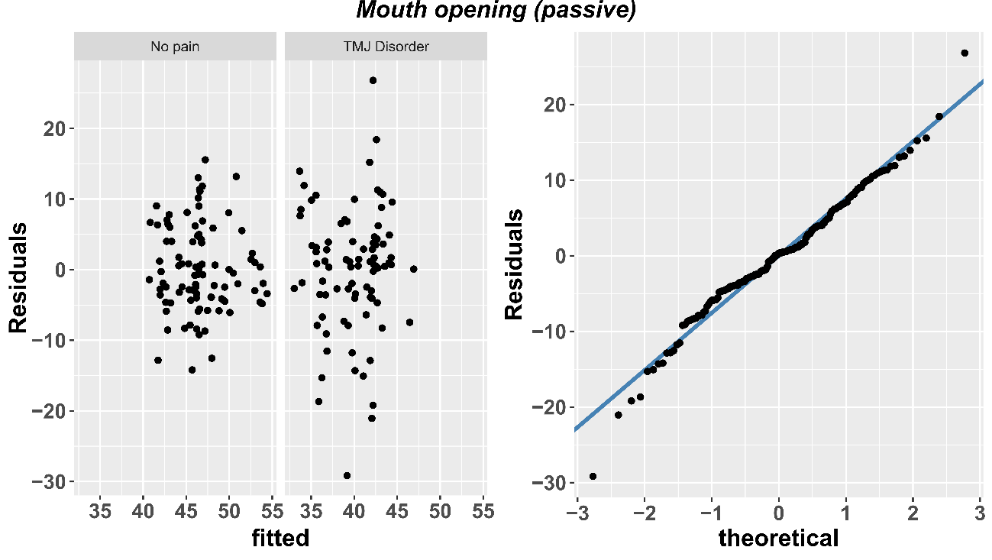


Supplementary Fig. 4. Residual plots for passive mouth opening.


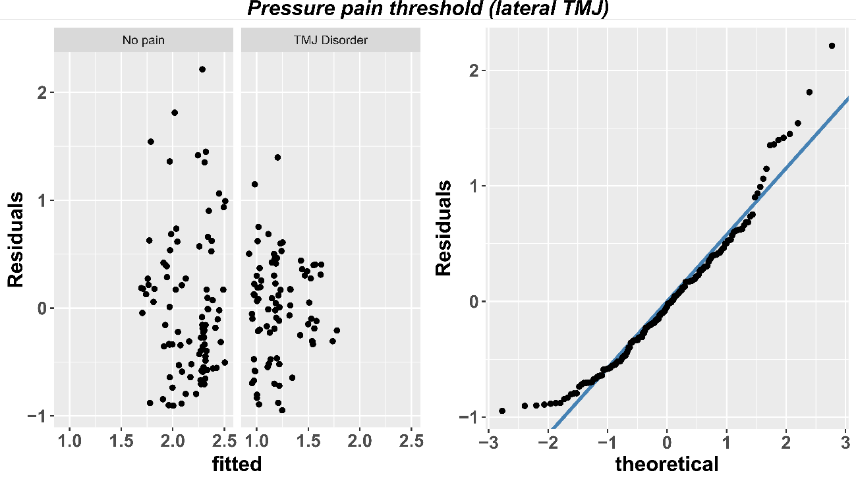


Supplementary Fig. 5. Residual plots for pressure pain threshold measured at the lateral of the temporomandibular joint.


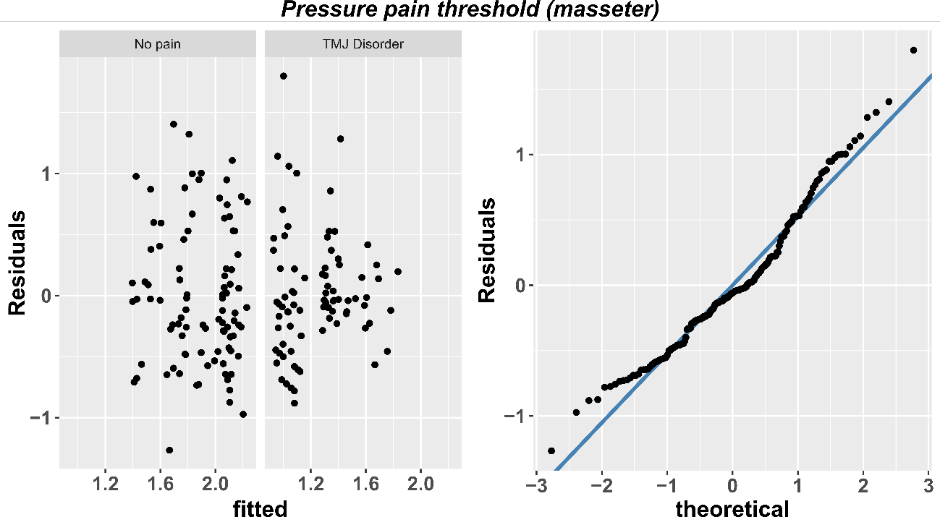


Supplementary Fig. 6. Residual plots for pressure pain threshold measured at the masseter muscle.


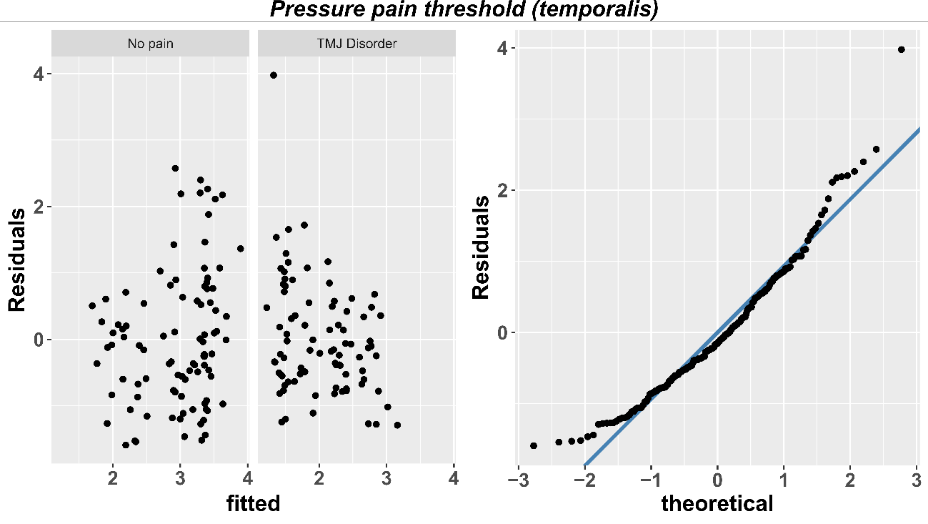


Supplementary Fig. 7. Residual plots for pressure pain threshold measured at temporalis muscle.


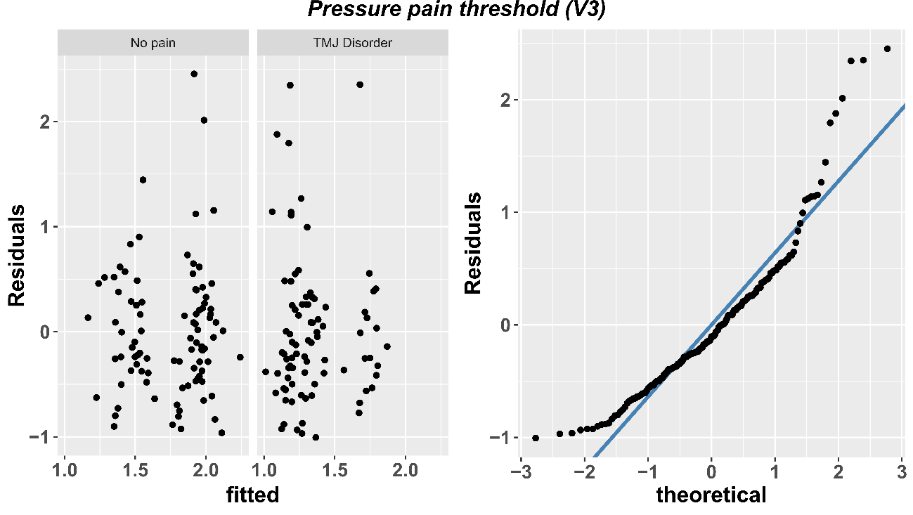


Supplementary Fig. 8. Residual plots for pressure pain threshold measured at the V3 of trigeminal nerve.


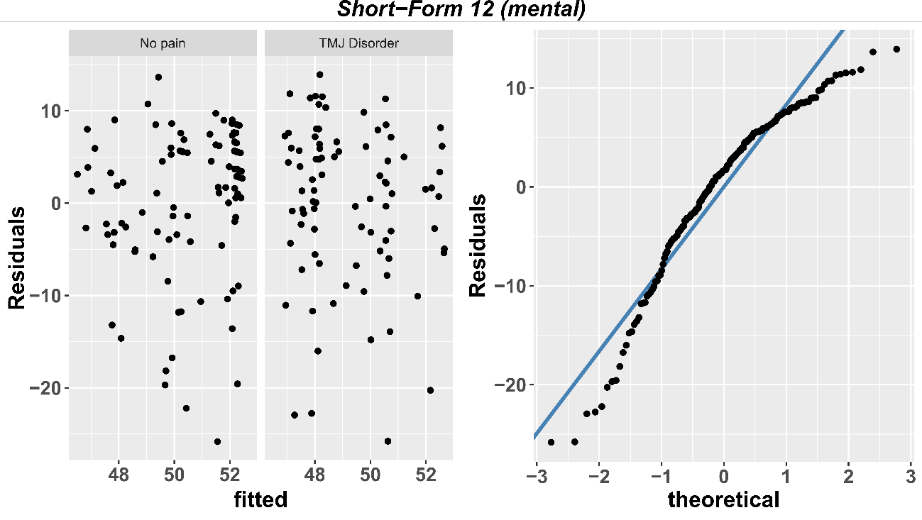


Supplementary Fig. 9. Residual plots for Short-Form 12 questionnaire mental subscale.


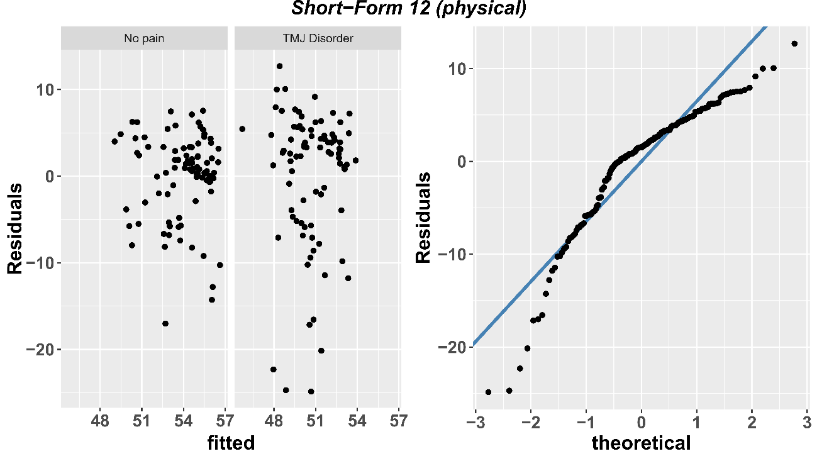


Supplementary Fig. 10. Residual plots for Short-Form 12 questionnaire physical subscale.


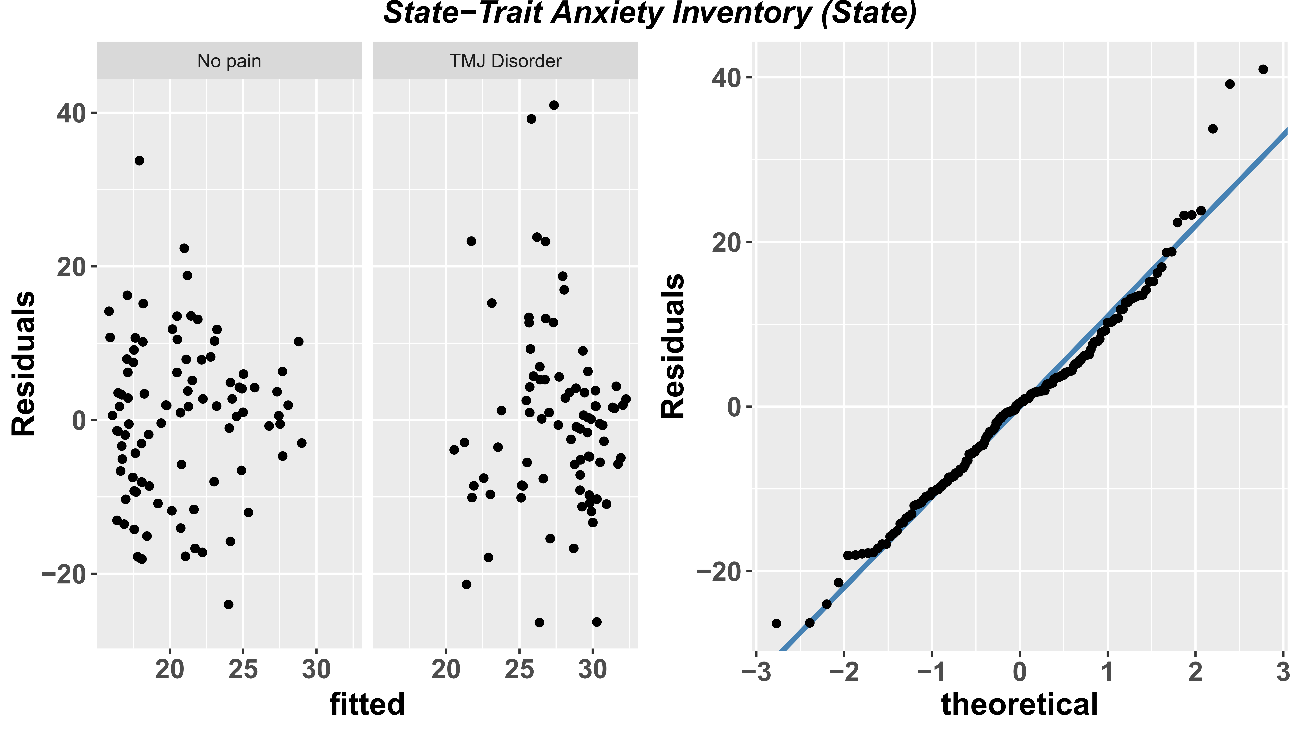


Supplementary Fig. 11. Residual plots for State-Trait Anxiety Inventory (state subscale).


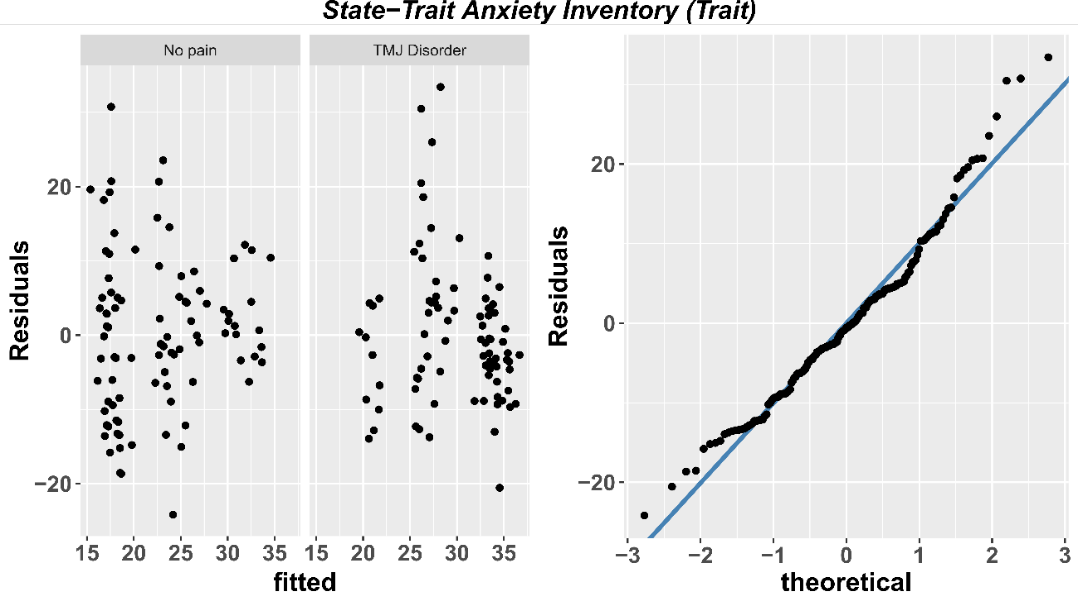


Supplementary Fig. 12. Residual plots for Short-Form 12 questionnaire mental subscale (trait subscale).

# Supplementary material 7. Full statistics of multiple parallel mediation model

********************* PROCESS for R Version 4.3.1 *********************

Written by Andrew F. Hayes, Ph.D. www.afhayes.com

Documentation available in Hayes (2022). www.guilford.com/p/hayes3

***********************************************************************

Model: 4

Y: ROM_active *(Active mouth opening)*

X: Pain *(Presence of temporomandibular joint disorder)*

M1: PPT_masseter *(PPT = Pressure pain threshold)*

M2: PPT_temporalis

M3: PPT_V3*(V3 =* *mandibular division of trigeminal nerve)*

M4: PPT_lateral_TMJ *(TMJ = temporomandibular joint)*

Covariates:

Age Weight Height Sex

Sample size: 179

Random seed: 932565

***********************************************************************

Outcome Variable: PPT_masseter

Model Summary:

*R* *R*-sq MSE *F*  *df*1 *df*2 *p*

0.6133 0.3762 0.2842 20.8645 5.0000 173.0000 <0.001

Model:

coeff se *t*  *p*  LLCI ULCI

constant 1.1231 0.2723 4.1238 0.0001 0.5855 1.6606

Pain −0.4510 0.0881 −5.1163 0.001 −0.6249 −0.2770

Age 0.0038 0.0034 1.1161 0.2659 −0.0029 0.0105

Weight 0.0034 0.0039 0.8634 0.3891 −0.0043 0.0111

Height 0.0022 0.0005 4.1050 0.0001 0.0011 0.0032

Sex 0.2125 0.1079 1.9684 0.0506 -0.0006 0.4255

***********************************************************************

Outcome Variable: PPT_temporalis

Model Summary:

*R* *R*-sq MSE  *F*  *df*1 *df*2  *p*

0.6060 0.3672 0.9020 20.0812 5.0000 173.0000 <0.001

Model:

coeff se  *t*  *p* LLCI ULCI

constant 2.1986 0.4852 4.5314 <0.001 1.2409 3.1562

Pain −0.5562 0.1570 −3.5418 0.0005 −0.8661 −0.2462

Age 0.0138 0.0061 2.2759 0.0241 0.0018 0.0258

Weight −0.0097 0.0070 −1.3922 0.1656 −0.0234 0.0040

Height 0.0054 0.0009 5.8145 <0.001 0.0036 0.0073

Sex 0.5353 0.1923 2.7837 0.0060 0.1557 0.9149

***********************************************************************

Outcome Variable: PPT_V3

Model Summary:

*R* *R*-sq MSE *F* *df*1 *df*2 *p*

0.4453 0.1983 0.4198 8.5592 5.0000 173.0000 <0.001

Model:

coeff se *t*  *p* LLCI ULCI

constant 1.6606 0.3310 5.0168 <0.001 1.0073 2.3140

Pain −0.2200 0.1071 −2.0533 0.0416 −0.4314 −0.0085

Age 0.0054 0.0041 1.2981 0.1960 −0.0028 0.0136

Weight −0.0071 0.0048 −1.4859 0.1391 −0.0164 0.0023

Height 0.0007 0.0006 1.0980 0.2737 −0.0006 0.0020

Sex 0.5547 0.1312 4.2276 <0.001 0.2957 0.8136

***********************************************************************

Outcome Variable: PPT_lateral_TMJ

Model Summary:

*R* *R*-sq MSE *F*  *df*1 *df*2 *p*

0.6680 0.4462 0.3418 27.8785 5.0000 173.0000 <0.001

Model:

coeff se  *t*  *p* LLCI ULCI

constant 1.4584 0.2987 4.8831 <0.001 0.8689 2.0479

Pain −0.7605 0.0967 −7.8671 <0.001 −0.9513 −0.5697

Age 0.0073 0.0037 1.9543 0.0523 -0.0001 0.0147

Weight 0.0015 0.0043 0.3546 0.7233 -0.0069 0.0100

Height 0.0013 0.0006 2.1681 0.0315 0.0001 0.0024

Sex 0.2982 0.1184 2.5191 0.0127 0.0645 0.5319

***********************************************************************

Outcome Variable: ROM_active

Model Summary:

*R*  *R*-sq MSE *F*   *df*1 *df*2  *p*

0.5370 0.2883 63.3212 7.6083 9.0000 169.0000 <0.001

Model:

coeff se *t*  *p* LLCI ULCI

constant 37.6573 4.4371 8.4870 <0.001 28.8981 46.4165

Pain −7.4955 1.5669 −4.7837 <0.001 −10.5887 −4.4024

PPT_masseter 0.4149 1.5750 0.2634 0.7925 −2.6942 3.5240

PPT_temporalis −0.7041 0.8574 −0.8212 0.4127 −2.3967 0.9885

PPT_V3 4.3194 1.3419 3.2189 0.0015 1.6704 6.9684

PPT_lateral_TMJ −2.2661 1.3717 −1.6520 0.1004 −4.9741 0.4418

Age −0.0274 0.0518 −0.5284 0.5979 −0.1297 0.0749

Weight 0.0533 0.0598 0.8907 0.3744 −0.0648 0.1715

Height −0.0175 0.0090 −1.9515 0.0526 −0.0353 0.0002

Sex 3.1108 1.6969 1.8332 0.0685 −0.2391 6.4608

***********************************************************************

Bootstrapping progress:

|>>>>>>>>>>>>>>>>>>>>>>>>>>>>>>>>>>>>>>>>>>>>>>>>>>>>>>>>>>>>>>| 100%

**************** DIRECT AND INDIRECT EFFECTS OF X ON Y ****************

Direct effect of X on Y:

effect se *t* *p* LLCI ULCI

−7.4955 1.5669 −4.7837 <0.001 −10.5887 −4.4024

Indirect effect(s) of X on Y:

Effect BootSE BootLLCI BootULCI

TOTAL 0.9777 1.0122 −0.8665 3.1192

PPT_masseter −0.1871 0.8684 −1.8631 1.5639

PPT_temporalis 0.3916 0.5251 −0.4558 1.6863

PPT_V3 −0.9502 0.5007 −2.0713 −0.1202

PPT_lateral_TMJ 1.7233 1.1468 −0.4426 4.0479

******************** ANALYSIS NOTES AND ERRORS ************************

Level of confidence for all confidence intervals in output: 95

Number of bootstraps for percentile bootstrap confidence intervals: 5000

# Supplementary material 8. Models comparison and diagnostic plots for beta-regression

Supplementary Table 5. Comparison of full and reduced models excluding pressure pain threshold and mental health predictors.

| Model | Df | LogLik | *Df* | χ^2^ | *p*-value | AIC |
| --- | --- | --- | --- | --- | --- | --- |
| Full model | 15 | 86.80 | - | - | - | −143.59 |
| Model without PPT outcomes | 11 | 85.21 | −4 | 3.17 | 0.53 | −148.42 |
| Model without PPT and mental outcomes | 8 | 84.57 | −3 | 1.27 | 0.74 | −153.15 |

Abbreviations: *Df*: degrees of freedom; AIC: Akaike Information Criterion; PPT: pressure pain threshold.


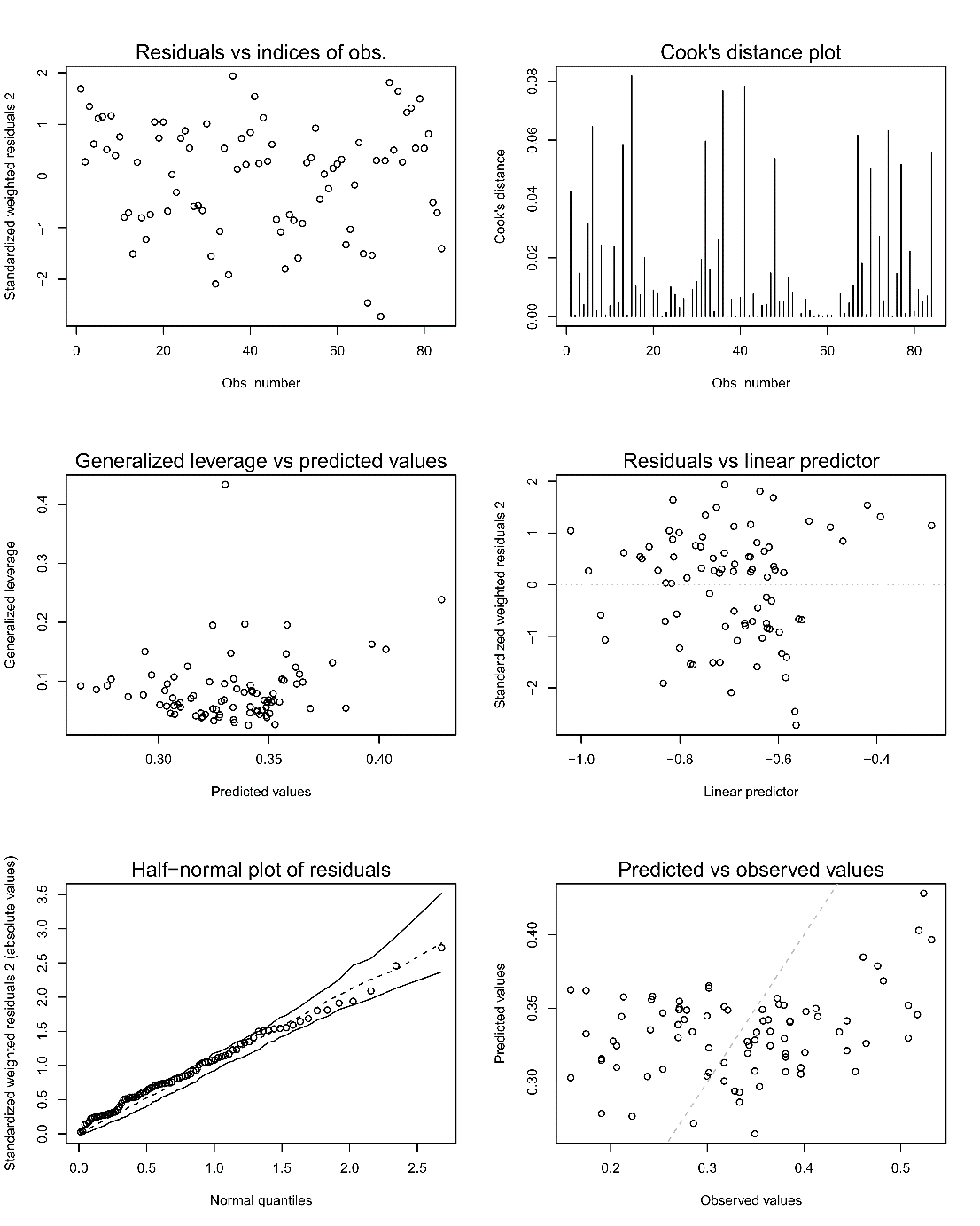


Supplementary Fig. 13. Diagnostic plots for the final beta-regression model for predicting Craniofacial Pain and Disability Inventory.
